# Supplementary material for: Restoring speech intelligibility for hearing aid users with deep learning
Source: Sci Rep. 2023 Feb 15;13:2719. doi: 10.1038/s41598-023-29871-8 (PMC9932078; doi:10.1038/s41598-023-29871-8)
Supplement: Supplementary file 1 — Supplementary Information. [file 41598_2023_29871_MOESM1_ESM.docx]

Restoring speech intelligibility for hearing aid users with deep learning

Peter Udo Diehl^1,2,*^, Yosef Singer^1^, Hannes Zilly^1^, Uwe Schönfeld^2^, Paul Meyer-Rachner^1^, Mark Berry^1^, Henning Sprekeler ^3,4,5^, Elias Sprengel^1^, Annett Pudszuhn^2^, Veit M. Hofmann^2^

* Corresponding author

^1^ Audatic, Berlin, Friedrichstr. 210, 10117 Berlin, Germany

^2^ Charité – Universitätsmedizin Berlin, corporate member of Freie Universität Berlin, Humboldt-Universität zu Berlin, and Berlin Institute of Health, Department of Otorhinolaryngology, Head and Neck Surgery, Campus Benjamin Franklin, Germany

^3^ Department for Electrical Engineering and Computer Science, Technische Universität Berlin, Berlin, Germany

^4^ Bernstein Center for Computational Neuroscience Berlin, Philippstr. 13, 10115 Berlin, Germany

^5^ Exzellenzcluster Science of Intelligence, Technische Universität Berlin, Marchstr. 23, 10587 Berlin, Germany

# Extended Data

Extended Data Table 1: Rating instructions for Mechanical Turk human raters

| Score | Includes | Excludes |
| --- | --- | --- |
| 5 – Excellent | Sample contains a clear voice/clear speech that is easily understandable | Excludes samples that have any background noise or distortion of the speech |
| 4 - Good | Sample contains clear voice with either minimal distortion or every minimal background noise. | Exclude samples that have significant background noises or obvious distortion of the speech (this would get a score of 3) |
| 3 - Fair | Sample contains voice that is either distorted or has significant background noise but the speech is still understandable without too much effort | Exclude samples that are so distorted/with so much background noise that words (even single words) are not understandable (this would get a score of 2). Also, if it is difficult to decide whether there is background noise or distortions it should be excluded (this would get a score of 4). |
| 2 - Poor | Sample contains speech where it’s possible to understand most of the words when concentrating or hearing the clip multiple times. There can be loud background noise and the voice can be heavily distorted such that it’s hard to understand but not impossible. | Excludes samples where the majority of words are not understandable (this would get a score of 1) or where a lot of noise is present but it is easy to understand all the words without too much effort (this would get a score of 3). |
| 1 – Bad | Samples in this category contain highly unpleasant noise, to the extent that speech is almost impossible to understand. Also choose this score if background noise is not as unpleasant but speech cannot be understood. | Exclude samples where most of words can be understood (this would get a score of 2). |


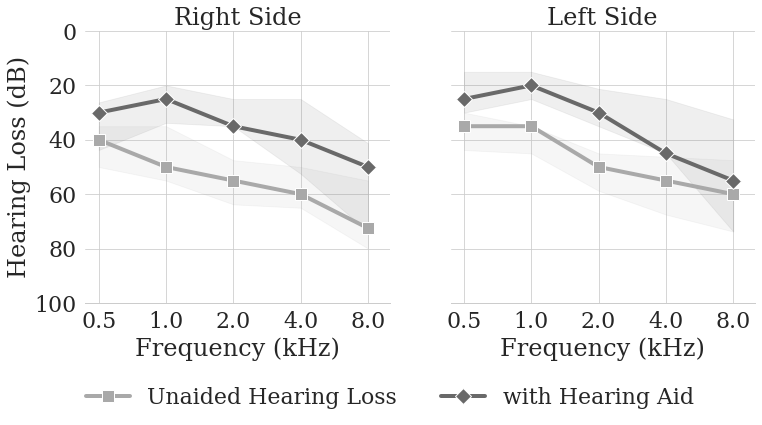


Extended Data Figure 1**. Median hearing loss of the subjects – aided and unaided.** Subjects used their existing fittings for the test, i.e. we did not re-fit the hearing aids. For the OLSA test, hearing impaired subjects listened with their hearing aids. Shading depicts quartiles.


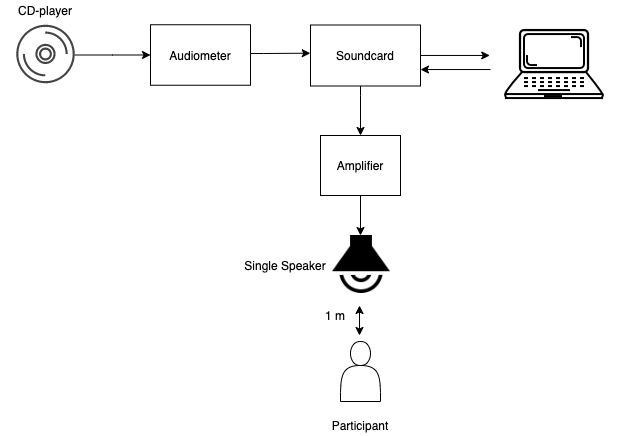


Extended Data Figure 2: **Testing setup for hearing impaired and normal hearing subjects.** We calibrated loudness levels to the position of a chair approximately 1m away from the speaker where subjects sat.


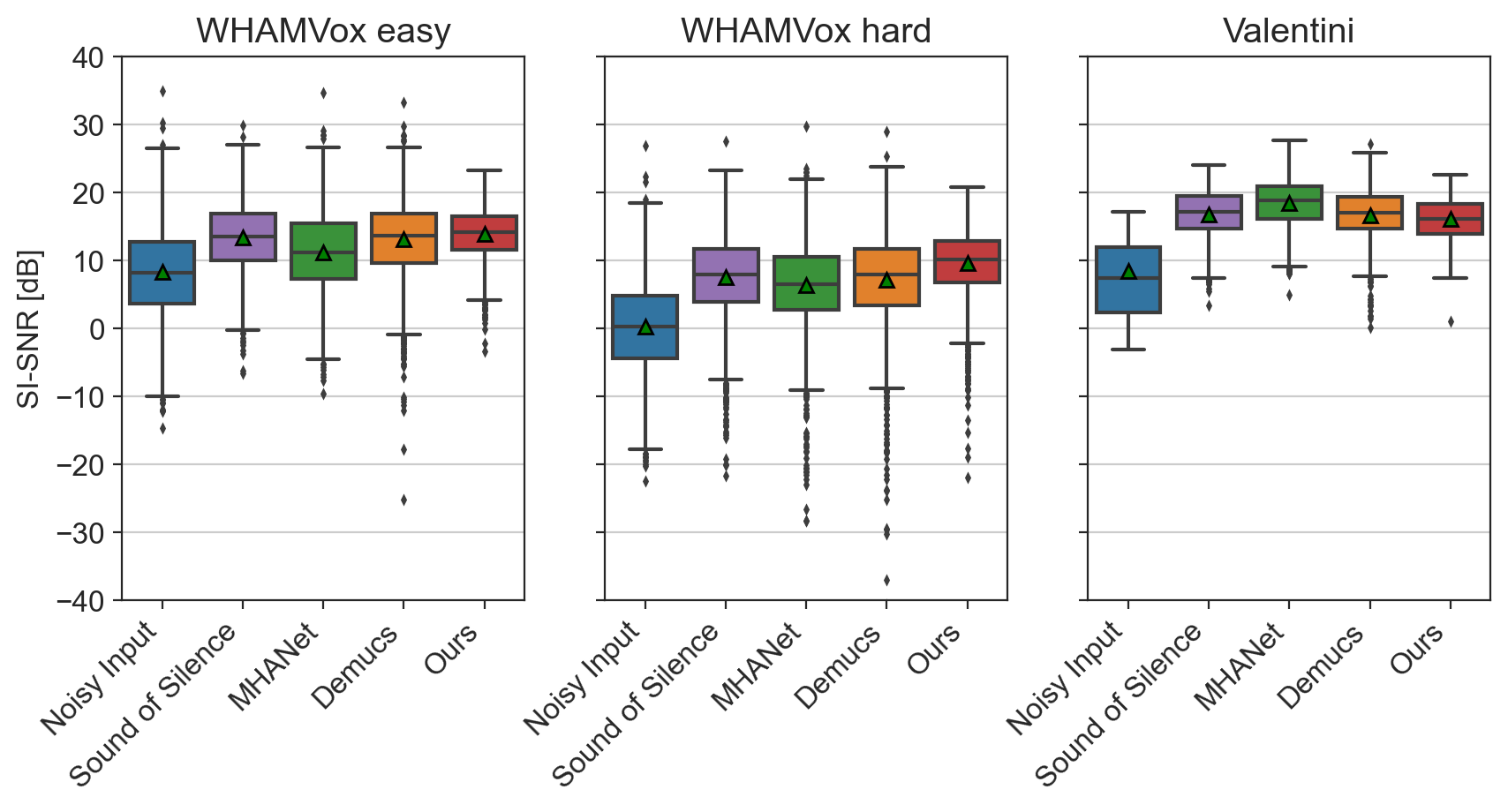
 Extended Data Figure 3. **SI-SNR comparison of state-of-the-art denoising methods**. Triangles denote the mean, the bar chart shows the 25%, 50% and 75% quartiles. Whiskers show the 1.5 times the interquartile range and diamonds denote outliers.
